# A Rare Case of Hereditary Pancreatitis Unveiling Systemic Lupus Erythematosus in a Young Female Patient

**DOI:** 10.7759/cureus.85209

**Published:** 2025-06-01

**Authors:** Shaheed Shaik, Shruti M Mundada, Siddharth Vatsi Reddy Thimmannagari, Srihitha Arra, Yashwanth Dora Borra

**Affiliations:** 1 General Medicine, Gandhi Medical College and Hospital, Hyderabad, IND; 2 General Internal Medicine, Gandhi Medical College and Hospital, Hyderabad, IND

**Keywords:** antinuclear antibody (ana), hereditary pancreatitis, “pancytopenia”, spink 1 mutation, systemic lupus erythematosus

## Abstract

Systemic lupus erythematosus (SLE) is a chronic autoimmune disease with varied clinical presentations, sometimes occurring in atypical presentations that cause a delay in diagnosis. Pancreatitis, though uncommon, may be an initial presentation of SLE and needs to be carefully evaluated to rule out other causes. We present the case of a 19-year-old woman who presented with vomiting and abdominal pain and was later diagnosed with hereditary pancreatitis following the detection of a heterozygous SPINK1 gene mutation. Additional evaluation showed systemic manifestations such as non-scarring alopecia, painful oral ulcers, pancytopenia, lupus hepatitis, and a highly positive autoimmune profile, in keeping with a new diagnosis of SLE. This case highlights the diagnostic challenge at the interface of genetic and autoimmune pancreatitis and reinforces the need for a thorough clinical, immunologic, and genetic evaluation in young patients with unexplained abdominal complaints.

## Introduction

Acute pancreatitis is an inflammatory disorder of the pancreas, marked by abdominal pain and increased levels of pancreatic enzymes. Although it is frequently seen in adults, often caused by gallstones or alcohol use, its presentation in younger individuals is uncommon and warrants a detailed evaluation for less typical causes. In cases of recurrent or severe pancreatitis among young patients, it is important to explore both hereditary and organic factors. Genetic mutations, such as those affecting the SPINK1 gene, may predispose individuals to pancreatitis by reducing the threshold for pancreatic inflammation, especially when other triggers are present. Additionally, organic conditions like primary hyperparathyroidism can lead to pancreatitis through elevated calcium levels, which activate pancreatic enzymes within the acinar cells [1].

Hereditary pancreatitis (HP) is a rare genetic disorder characterized by recurrent episodes of acute or chronic pancreatitis, typically manifesting before the age of 20. It is most commonly linked to pathogenic mutations in the genes encoding protease serine 1 (PRSS1), serine peptidase inhibitor Kazal type 1 (SPINK1), and the cystic fibrosis transmembrane conductance regulator (CFTR) [2]. Among these, SPINK1 mutations act as disease modifiers rather than primary causative factors and generally require an additional trigger, such as environmental or autoimmune influences, to manifest clinically [3].

Systemic lupus erythematosus (SLE) is a chronic multisystem autoimmune disorder that can involve the pancreas among its diverse range of affected organs [4]. Although pancreatitis is an uncommon manifestation of SLE, its presence should not be overlooked, particularly when accompanied by other autoimmune features [5]. Diagnosing pancreatitis as an initial presentation of SLE is often challenging and requires careful exclusion of more common causes such as gallstones, alcohol use, or drug-induced injury [6].

This case report describes a young woman presenting with acute necrotizing pancreatitis who was ultimately diagnosed with both SPINK1-related hereditary pancreatitis and active SLE, underscoring the importance of integrating autoimmune and genetic evaluations in atypical and severe cases of pancreatitis.

## Case presentation

A 19-year-old female student presented to the emergency department with a 20-day history of severe, deep, boring abdominal pain localized to the epigastric and left hypochondriac regions. The pain had a sudden onset, radiated to the back, worsened with food intake, and was partially relieved by analgesics. She also experienced three to four episodes of non-bilious, non-projectile vomiting daily, consisting of semi-digested food particles. Over the same period, the patient reported an unintentional weight loss of approximately 4 kg. There was no history of hematemesis, melena, bloating, heartburn, abdominal distension, constipation, steatorrhea, or any changes in bowel habits. She was admitted and managed symptomatically for one week and subsequently discharged.

However, the patient returned one week later with recurrent abdominal pain of similar character and severity. This second presentation prompted a more extensive evaluation. Additional history revealed that three months prior, she had developed elevated erythematous lesions over the palms and soles. These were non-pruritic and non-tender, resolving spontaneously with ulceration and residual hyperpigmentation. She had also experienced painful mucosal ulcers over the hard palate and buccal mucosa. Over the past two months, the patient experienced intermittent low-grade fever without associated chills or rigors, along with generalized weakness, loss of appetite, and gradually worsening shortness of breath, classified as grade 2 on the modified Medical Research Council (mMRC) Dyspnea Scale. This was associated with easy fatigability but not with orthopnea or paroxysmal nocturnal dyspnea (PND). She denied any chest pain, palpitations, pedal edema, jaundice, or urinary symptoms. Her menstrual cycles were regular, with no dysmenorrhea. There was no relevant family history of similar complaints. Table 1 summarizes the timeline of the patient’s symptoms, hospital admissions, investigations, and treatment, providing an overview of the clinical course leading to the final diagnosis and management.

**Table 1:** Chronological summary of symptoms, diagnostic workup, and management mMRC: modified Medical Research Council; SLE: systemic lupus erythematosus

Time Frame	Event / Symptom
3 months before admission	Red, raised lesions on palms and soles (non-itchy, non-tender); healed with ulcers and dark pigmentation; painful mouth ulcers on hard palate and cheeks
2 months before admission	Off-and-on low-grade fever without chills or rigors; general weakness; poor appetite and unintentional weight loss (~4 kg)
1 month before admission	Gradual onset of breathlessness (mMRC Dyspnea Scale grade 2); easy fatigue; no shortness of breath while lying down or sudden breathlessness at night
First hospital visit	Severe abdominal pain for 20 days (epigastric and left upper abdomen), radiating to the back; worsened after meals; 3–4 episodes/day of non-bilious vomiting
First hospitalization	Admitted to the medical ward; underwent basic investigations and received supportive care; discharged after 7 days
One week after discharge	Abdominal pain recurred; readmitted for further evaluation
Day 1 of second admission	Abdominal ultrasound performed
Day 2 of second admission	Contrast-enhanced CT scan of the abdomen done
Day 6 of second admission	Fibroscan showed fatty liver and increased stiffness, bone marrow biopsy also done
Day 10 of second admission	Results of immunological and genetic testing became available
Day 11 of second admission	Final diagnosis: SLE with hereditary pancreatitis due to SPINK1 mutation
Day 12 of second admission	Started on high-dose intravenous steroids (methylprednisolone) and cyclophosphamide

**Figure 1:**
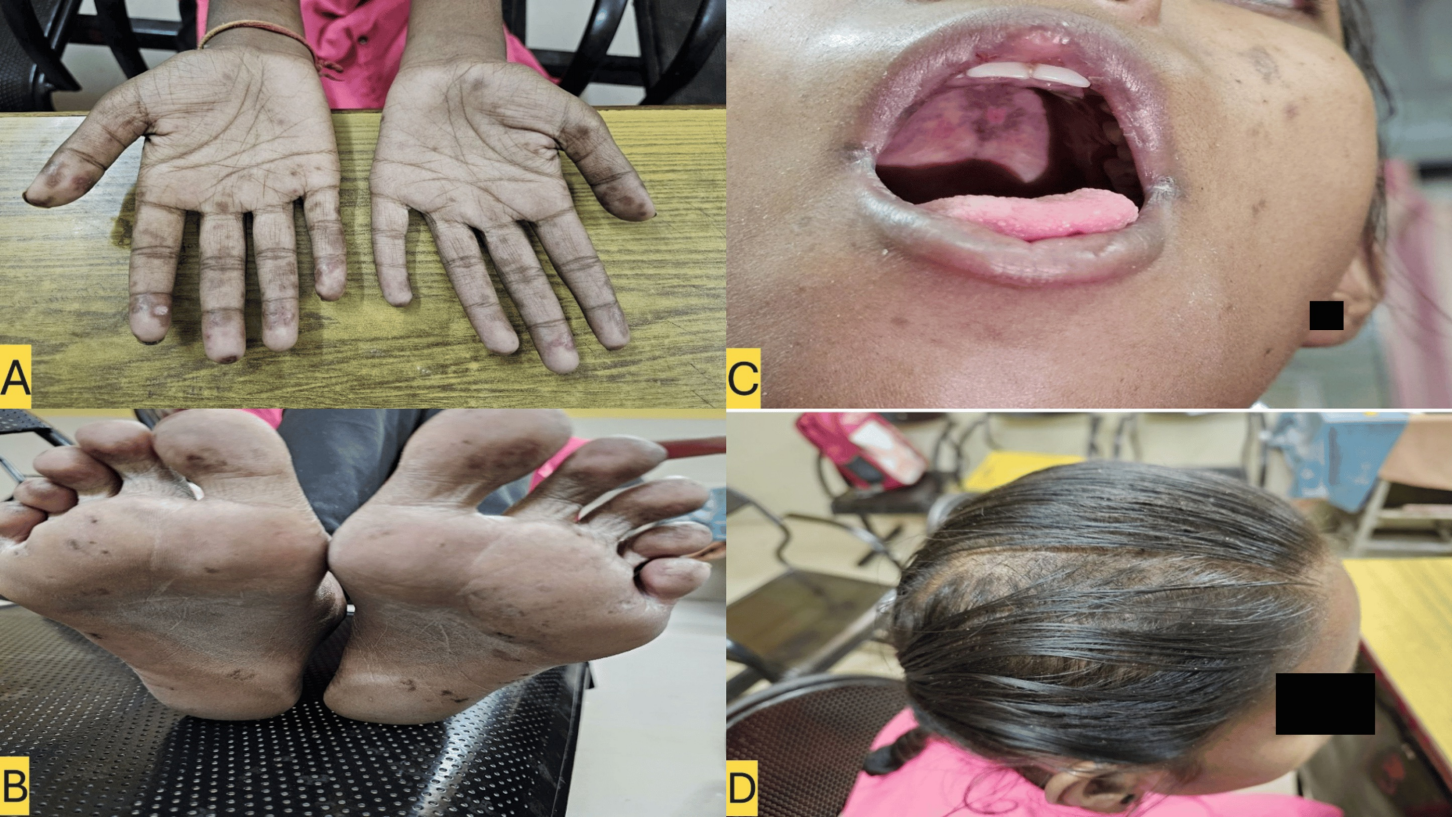
Panel A and B shows hyperpigmented macules and pitted scars on the palms and soles. Panel C shows angular cheilitis, and healing ulcers on the palate. Panel D shows non-scarring alopecia

On physical examination, the patient appeared pale but was alert and oriented. Her body mass index (BMI) was 19.6 kg/m². Hyperpigmented macules and pitted scars were observed on the palms (Figure 1A) and soles (Figure 1B). Healing ulcers were noted on the palate, along with angular cheilitis (Figure 1C), and non-scarring alopecia was evident (Figure 1D). Vital signs were within normal limits. Abdominal examination revealed tenderness in the epigastric and left hypochondriac regions, without signs of peritonitis or organomegaly. Examination of the other systems was unremarkable.

Laboratory investigations revealed severe anemia with an initial hemoglobin of 6.1 g/dL. Serum iron, total iron-binding capacity (TIBC), and transferrin saturation were within normal limits, while serum ferritin was markedly elevated, suggesting anemia of chronic disease. Serum vitamin B12 levels were significantly elevated, possibly due to inflammation-related release, with no history of exogenous vitamin B12 supplementation. Elevated serum lactate dehydrogenase (LDH), along with a positive direct Coombs test and a negative indirect Coombs test, indicated immune-mediated hemolysis. Peripheral blood smear showed anisopoikilocytosis, with predominantly normocytic and normochromic red cells and no fragmented cells. Bone marrow biopsy demonstrated hypocellularity with reduced erythropoiesis and both normoblastic and micronormoblastic maturation, suggestive of autoimmune-mediated marrow suppression.

The patient underwent sequential laboratory testing during the second admission, which revealed a general trend of improvement in hematologic, hepatic, and renal function. As seen in Table 2, key hematologic and biochemical parameters measured on four separate occasions highlight the patient’s response to therapy. Similarly, Table 3 shows the biochemical profile specifically aimed at evaluating hematinic status and excluding a potential hemolytic component.

**Table 2:** Dynamic trends in key laboratory values during clinical management AST: aspartate transaminase; ALT: alanine transaminase; WBC: white blood cell count

Parameter	Reference range	Day 1	Day 4	Day 9	Day 16
Hemoglobin (g/dL)	12–16	6.1	6.3	6.9	8.8
Total WBC (×10³/µL)	4.0–11.0	1.7	1.2	2.6	3.4
Platelets (×10⁵/µL)	1.5–4.5	1.36	1.29	1.22	1.76
Urea (mg/dL)	15–40	29	27	12	15
Creatinine (mg/dL)	0.6–1.3	1.1	1.1	0.6	0.62
Sodium (mEq/L)	135–145	133	134	136	137
Potassium (mEq/L)	3.5–5.0	4	4	4	3.6
Chloride (mEq/L)	98–107	104	104	100	105
Total bilirubin (mg/dL)	0.3–1.2	1.8	1.7	1.7	1.2
Direct bilirubin (mg/dL)	0–0.3	1.2	1.0	1.0	0.8
Indirect bilirubin (mg/dL)	0.2–0.9	0.6	0.7	0.7	0.4
AST (U/L)	<40	799	548	332	231
ALT (U/L)	<40	152	112	67	48
Alkaline phosphatase (U/L)	44–147	232	234	332	249
Total protein (g/dL)	6.0–8.3	5.8	5.4	5.3	6.6
Albumin (g/dL)	3.5–5.0	2.6	2.4	2.3	3
Globulin (g/dL)	2.0–3.5	3	3	3	3.6

**Table 3:** Biochemical parameters related to hematinic status and hemolysis workup TIBC: total iron binding capacity; TSAT: transferrin saturation; LDH: lactate dehydrogenase

Parameter	Reference Range	Value
Serum iron	35–145	106 µg/dL
TIBC	240–450	338 µg/dL
TSAT	20–50	28%
Serum ferritin	30–400	551.6 ng/mL
Vitamin B12	200–900	>1500 pg/mL
Serum LDH	140–280	421 U/L
Direct Coombs test	Negative	Positive
Indirect Coombs test	Negative	Negative

**Figure 2:**
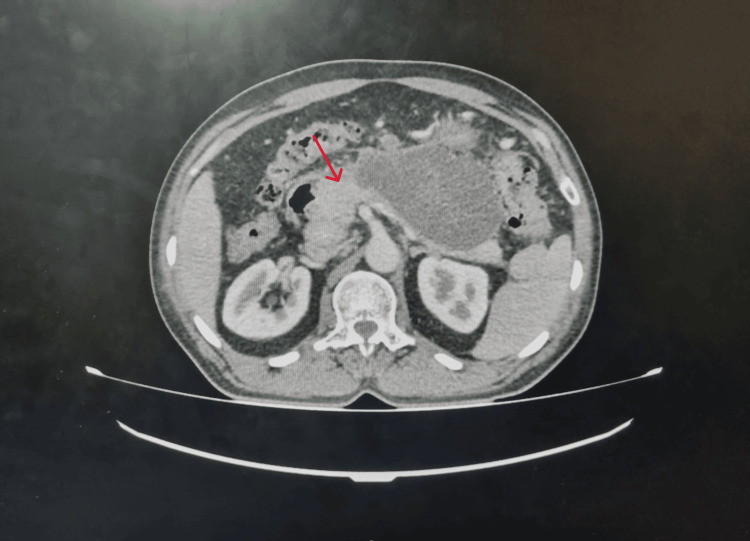
Contrast-enhanced computed tomography (CECT) of the abdomen showing an enlarged pancreas with peripancreatic inflammation and areas of necrosis (indicated by a red arrow), consistent with acute necrotizing pancreatitis.

Pancreatic enzyme testing showed an amylase level of 130 U/L and a markedly elevated lipase level of 753 U/L. An initial abdominal ultrasonography revealed a bulky head, body, and tail of the pancreas with normal echotexture; however, visualization was significantly limited due to bowel gas interference. The scan also showed Grade 2 fatty liver, with no evidence of gallstones. Given the persistent severe abdominal pain, recurrent vomiting, and suboptimal ultrasound findings, an early contrast-enhanced computed tomography (CECT) was warranted. The CECT demonstrated a swollen pancreas with peripancreatic inflammation and areas of necrosis (Figure 2), consistent with acute necrotizing pancreatitis. The modified computed tomography severity index (CTSI) score was 4/10, indicating mild pancreatitis. There were no signs of pancreatic calcifications or ductal dilatation.

A liver Fibroscan performed on day 6 of the second admission revealed a liver stiffness measurement of 7.3 kPa, indicating mild hepatic fibrosis (F1-F2), and a median ultrasound attenuation parameter (UAP) of 315 dB/m, consistent with severe hepatic steatosis.

Given the patient's young age and recurrent episodes of pancreatitis, common causes such as alcohol use, drug-induced pancreatitis, and hypertriglyceridemia were definitively ruled out. This exclusion supported a hereditary etiology. Additionally, the presence of systemic features, including pancytopenia, significant fatigue, alopecia, painful oral ulcers, and intermittent low-grade fever raised concern for an underlying autoimmune disorder. These clinical findings prompted a comprehensive autoimmune and genetic workup.

The autoimmune profile revealed a highly positive antinuclear antibody (ANA) with a nuclear speckled pattern at a 1:10,000 titre. Additional serological markers were strongly positive for anti-nuclear ribonucleoprotein (anti-nRNP), anti-Smith (anti-Sm) antibodies, anti-double-stranded DNA (anti-dsDNA), nucleosomes, and beta-2 glycoprotein antibodies. Complement levels were low, with C3 at 36 mg/dL and C4 less than 5 mg/dL. The erythrocyte sedimentation rate (ESR) was markedly elevated at 120 mm/hour in the first hour. Urinalysis showed 1+ proteinuria, with a 24-hour urinary protein excretion of 450 mg/day, suggestive of renal involvement in the context of lupus.

To investigate a potential hereditary component contributing to pancreatitis, genetic testing was performed. This identified a heterozygous mutation in the SPINK1 gene, while the PRSS1 gene was normal. Viral marker screening, including hepatitis and HIV, was negative, and serum IgG4 was within normal limits at 0.7 g/L.

These findings collectively supported a diagnosis of SLE coexisting with SPINK1-related hereditary pancreatitis, providing a comprehensive explanation for the patient’s multifaceted clinical presentation. Based on clinical, biochemical, radiological, genetic, and immunological findings, the final diagnosis was hereditary pancreatitis precipitated by active SLE with associated pancytopenia, lupus hepatitis, proteinuria, chilblain lupus, and non-scarring alopecia. The patient’s SLE scores were as follows: American College of Rheumatology/European League Against Rheumatism (ACR/EULAR) score of 25; Systemic Lupus International Collaborating Clinics (SLICC) criteria with 5 clinical and 5 immunological points; and a Systemic Lupus Erythematosus Disease Activity Index 2000 (SLEDAI-2K) score of 12.

The patient received pulse therapy with methylprednisolone followed by oral prednisolone at 1 mg/kg, along with cyclophosphamide pulses of 500 mg and daily hydroxychloroquine 200 mg. Cyclophosphamide was chosen due to the presence of significant hematologic and hepatic involvement, fulfilling criteria for moderate-to-severe organ-threatening SLE. This approach aimed to induce remission rapidly and preserve organ function. Supportive treatment for pancreatitis included careful fluid replacement and nutritional optimization.

The patient showed gradual clinical improvement during hospitalization with resolution of abdominal symptoms and normalization of laboratory parameters. She was discharged in stable condition with instructions for follow-up in the gastroenterology and rheumatology outpatient departments. However, she demonstrated poor follow-up adherence after discharge, which limited further monitoring and evaluation. This highlights a key learning point regarding the importance of structured follow-up, especially in patients with chronic or overlapping autoimmune and genetic conditions.

## Discussion

This case highlights the rare and diagnostically challenging coexistence of hereditary pancreatitis and SLE in a young woman. The patient's initial presentation with acute pancreatitis prompted standard evaluation, which later evolved into a broader workup due to persistent pancytopenia, oral ulcers, alopecia, and constitutional symptoms. These features raised suspicion for an underlying autoimmune process. The patient fulfilled both ACR/EULAR and SLICC classification criteria for SLE and had an SLEDAI-2K score of 12, consistent with moderate disease activity [7]. Hematologic involvement was supported by peripheral smear and bone marrow biopsy findings of hypocellularity with normoblastic maturation, consistent with immune-mediated marrow suppression typically seen in SLE [8].

The coexistence of a SPINK1 mutation and active SLE suggests a two-hit model, wherein a genetic predisposition was unmasked or exacerbated by immune-mediated inflammation. SPINK1 mutations are associated with hereditary pancreatitis, but they often require an environmental or immune-mediated trigger to manifest clinically [3,9]. In this case, active SLE likely served as a precipitating second hit that unmasked the underlying genetic predisposition and triggered necrotizing pancreatitis. This interplay highlights the importance of considering both autoimmune and hereditary causes in young female patients presenting with abdominal pain, especially when traditional risk factors are absent, reinforcing the need for a broad and stepwise diagnostic approach guided by clinical findings. Lupus-related hepatic involvement was also evident, characterized by elevated transaminases in the absence of autoimmune hepatitis markers such as anti-smooth muscle and anti-liver-kidney microsomal (anti-LKM) antibodies [10]. Fibroscan findings of mild fibrosis and extensive hepatic steatosis pointed to both metabolic and immune contributions [11].

Although pancreatitis is an uncommon manifestation of SLE, it is a recognized complication that may result from small vessel vasculitis, immune complex deposition, or direct immune cell infiltration [4,5]. In rare instances, this immune-mediated vascular injury may impair pancreatic perfusion and lead to necrotizing pancreatitis (NP), particularly when compounded by genetic predispositions such as SPINK1 mutations [12]. The dual pathology in this case underscores the importance of maintaining a broad differential diagnosis and using a multimodal approach that integrates clinical, serological, and genetic data [13]. Furthermore, while the patient showed clinical improvement and was discharged after symptom resolution, her poor post-discharge follow-up limited ongoing assessment. This highlights a key learning point: structured follow-up planning is essential, especially in complex, multisystemic conditions where long-term monitoring and management are critical.

## Conclusions

This case underscores the importance of maintaining a broad differential diagnosis when evaluating acute pancreatitis in young patients, particularly in the absence of common risk factors. Hereditary causes should be considered when the presentation is early-onset, recurrent, or unexplained, while autoimmune etiologies like SLE should be suspected in the presence of systemic features such as pancytopenia, oral ulcers, alopecia, or constitutional symptoms. The coexistence of SPINK1-related hereditary pancreatitis and active SLE in this patient illustrates how overlapping genetic and immune mechanisms can complicate the clinical picture, influence diagnostic priorities, and guide treatment decisions.

This case also underscores the importance of structured follow-up, as poor post-discharge monitoring limited long-term assessment and highlights the need for improved patient education and continuity of care. It also reinforces the need for clinical vigilance and supports the integration of genetic and autoimmune testing when evaluating atypical or severe pancreatitis in young individuals.
